# Supplementary material for: Protectivity of COVID-19 Vaccines and Its Relationship with Humoral Immune Response and Vaccination Strategy: A One-Year Cohort Study
Source: Vaccines (Basel). 2022 Jul 25;10(8):1177. doi: 10.3390/vaccines10081177 (PMC9330104; doi:10.3390/vaccines10081177)
Supplement: Supplementary file 1 [file vaccines-10-01177-s001.zip › vaccines-1784665-supplementary.pdf]

Table S1. Inter-dose intervals by vaccine cohorts.

**Vaccine cohorts**		**Inter-dose intervals (days)**				**Interval since the dose-1 (days) ***
		**between dose- 1–2**	**between dose- 2–3**	**between dose- 3–4**	**between dose- 4–5**	
2-dose-CV	Mean	38.75				316.57
	Median	29.00				363.00
	Minimum	13.00				137.00
	Maximum	229.00				376.00
3-dose-CV	Mean	44.13	155.71			331.26
	Median	30.00	158.00			367.00
	Minimum	14.00	29.00			136.00
	Maximum	262.00	341.00			378.00
4-dose-CV	Mean	30.13	130.46	167.33		358.13
	Median	29.00	140.00	160.00		367.00
	Minimum	28.00	8.00	126.00		295.00
	Maximum	36.00	188.00	266.00		377.00
2-dose-BNT	Mean	51.19				209.90
	Median	36.50				214.00
	Minimum	19.00				83.00
	Maximum	230.00				398.00
3-dose-BNT	Mean	35.55	169.25			234.73
	Median	33.00	165.50			228.00
	Minimum	19.00	92.00			144.00
	Maximum	105.00	397.00			461.00
2-dose-CV+1-dose-BNT	Mean	31.53	162.96			349.19
	Median	28.50	147.50			368.00
	Minimum	19.00	53.00			164.00
	Maximum	212.00	335.00			380.00
3-dose-CV+1-dose-BNT	Mean	29.00	142.81	156.72		366.81
	Median	28.00	139.00	169.00		369.00
	Minimum	28.00	134.00	44.00		351.00
	Maximum	34.00	181.00	198.00		378.00
2-dose-CV+2-dose-BNT	Mean	29.44	143.34	103.05		359.91
	Median	28.00	140.00	98.00		368.00
	Minimum	14.00	30.00	21.00		173.00
	Maximum	122.00	295.00	292.00		420.00
2-dose-CV+3-dose-BNT	Mean	29.28	139.71	55.10	128.85	369.02
	Median	28.00	139.00	55.00	130.00	370.00
	Minimum	10.00	42.00	25.00	23.00	279.00
	Maximum	147.00	219.00	99.00	152.00	439.00

CV: CoronaVac™ BNT:Comirnaty®

* The time period in days between the first day of vaccination and the day when the blood sample was taken

Table S2. Incidence of adverse events in vaccine-dose subgroups.

Adverse events	Doses n (colon %)				
	I-dose (n=942)	II-dose (n=942)	III-dose (n=814)	IV-dose (n=466)	V-dose (n=92)
Rush	31 (3.3)	28(3.0)	41(5.0)	22(4.7)	1 (1.1)
Pain at the injection site	281(29.8)	273(29.0)	369(45.3)	229(49.1)	22 (24.0)
Swelling	33(3.5)	29(3.1)	55(6.8)	32(6.8)	0
Itching	10(1.1)	9(1.0)	17(2.1)	11(2.4)	0
Hypoesthesia	2(0.2)	2(0.2)	8(1.0)	7(1.5)	0
Induration	31(3.3)	30(3.2)	46(5.7)	26(5.6)	1(1.1)
Numbness in the vaccinated arm	30 (3.2)	26(2.8)	40(4.9)	24(5.1)	1(1.1)
Weakness	147(15.6)	138(14.6)	191(23.4)	130(28.0)	5(5.4)
Fatigue	120(12.7)	112(11.9)	175(21.5)	111(24.0)	6(6.5)
Fever	37(3.9)	32(3.4)	74(9.1)	57(12.2)	6(6.5)
Tremor	30(3.2)	28(3.0)	43(5.3)	33(7.0)	3(3.2)
Chest pain	10(1.1)	6(0.6)	13(1.6)	5(1.1)	0
Diarrhoea	9(1.0)	3(0.3)	5(0.6)	6(1.2)	1(1.1)
Nausea	18(1.9)	11(1.2)	14(1.7)	4(0.9)	1(1.1)
Vomiting	3(0.3)	4(0.4)	6(0.7)	4(0.9)	1(1.1)
Headache	93(9.9)	78(8.3)	114(14.0)	74(15.9)	7(7.7)
Dizziness	35(3.7)	32(3.4)	50(6.1)	27(5.8)	3(3.2)
Vertigo	20(2.1)	15(1.6)	28(3.4)	21(4.5)	2(2.1)
Myalgia	69(7.3)	62(6.6)	127(15.6)	85(18.2)	2(2.1)
Back pain	43(4.6)	39(4.1)	78(9.6)	48(10.3)	2(2.1)
Joint pain	54(5.7)	55(5.8)	97(12.0)	73(15.7)	4(4.3)
Cough	6(0.6)	4(0.4)	7(0.8)	5(1.1)	0
Sore throat	7(0.7)	7(0.7)	11(1.3)	6(1.3)	1(1.1)
Dyspnoea	13(1.4)	7(0.7)	15(1.8)	9(1.9)	0
Papule	0	0	0	0	0
Abdominal pain	3(0.3)	3(0.3)	4(0.5)	1(0.2)	0
Loss of appetite	9(1.0)	7(0.7)	9(1.1)	8(1.8)	1(1.1)
Palpitation	13(1.4)	10(1.1)	7(0.8)	6(1.3)	0
Anosmia	6(0.6)	1(0.1)	3(0.4)	1(0.2)	0
Loss of taste	2(0.2)	0	1(0.1)	1(0.2)	0
Rash	1(0.1)	0	1(0.1)	1(0.2)	0
Numbness in the tongue	5(0.5)	1(0.1)	1(0.1)	0	0
Fainting	3(0.3)	1(0.1)	1(0.1)	1(0.2)	0
Increased blood pressure	12(1.3)	9(1.0)	6(0.7)	2(0.4)	0
Decreased blood pressure	3(0.3)	3(0.3)	0	2(0.4)	0
Urticaria	2(0.2)	0	1(0.1)	0	0
Anaphylaxis	1(0.1)	1(0.1)	0	0	0
Metallic taste in the tongue	1(0.1)	1(0.1)	1(0.1)	1(0.2)	1(1.1)
Lymphadenopathy	1(0.1)	1(0.1)	3(0.4)	2(0.4)	1(1.1)
